# The Neuroanatomical Basis for Posterior Superior Parietal Lobule Control Lateralization of Visuospatial Attention

**DOI:** 10.3389/fnana.2016.00032

**Published:** 2016-03-24

**Authors:** Yan Wu, Jiaojian Wang, Yun Zhang, Dingchen Zheng, Jinfeng Zhang, Menglin Rong, Huawang Wu, Yinyan Wang, Ke Zhou, Tianzi Jiang

**Affiliations:** ^1^Key Laboratory for NeuroInformation of the Ministry of Education, School of Life Science and Technology, University of Electronic Science and Technology of ChinaChengdu, China; ^2^Beijing Neurosurgical Institute, Capital Medical UniversityBeijing, China; ^3^State Key Laboratory of Brain and Cognitive Science, Institute of Biophysics, Chinese Academy of SciencesBeijing, China; ^4^Brainnetome Center, Institute of Automation, Chinese Academy of SciencesBeijing, China; ^5^National Laboratory of Pattern Recognition, Institute of Automation, Chinese Academy of SciencesBeijing, China; ^6^The Queensland Brain Institute, University of QueenslandBrisbane, QLD, Australia; ^7^CAS Center for Excellence in Brain Science, Institute of Automation, Chinese Academy of SciencesBeijing, China

**Keywords:** transcranial magnetic stimulation, anatomical connectivity, fronto-parietal network, posterior parietal cortex, superior parietal lobule

## Abstract

The right hemispheric dominance in visuospatial attention in human brain has been well established. Converging evidence has documented that ventral posterior parietal cortex (PPC) plays an important role in visuospatial attention. The role of dorsal PPC subregions, especially the superior parietal lobule (SPL) in visuospatial attention is still controversial. In the current study, we used repetitive transcranial magnetic stimulation (rTMS) and diffusion magnetic resonance imaging (MRI) techniques to test the role of posterior SPL in visuospatial attention and to investigate the potential neuroanatomical basis for right hemisphere dominance in visuospatial function. Transcranial magnetic stimulation (TMS) results unraveled that the right SPL predominantly mediated visuospatial attention compared to left SPL. Anatomical connections analyses between the posterior SPL and the intrahemispheric frontal subregions and the contralateral PPC revealed that right posterior SPL has stronger anatomical connections with the ipsilateral middle frontal gyrus (MFG), with the ipsilateral inferior frontal gyrus (IFG), and with contralateral PPC than that of the left posterior SPL. Furthermore, these asymmetric anatomical connections were closely related to behavioral performances. Our findings indicate that SPL plays a crucial role in regulating visuospatial attention, and dominance of visuospatial attention results from unbalanced interactions between the bilateral fronto-parietal networks and the interhemispheric parietal network.

## Introduction

Cerebral asymmetry, which has been characterized by both its functions and its connections, is a fundamental property of the human brain and a marker of successful development (Liu et al., 2009; Bishop, 2013). The left hemisphere of the human brain preferentially mediates language ability, whereas the right hemisphere preferentially mediates visuospatial abilities (Weintraub and Mesulam, 1987; Bookheimer, 2002; Cai et al., 2013; Corballis, 2014). Many previous clinical, neuropsychological and transcranial magnetic stimulation (TMS) studies have revealed that visuospatial attention was primarily controlled by the right ventral posterior parietal cortex (PPC; Arrington et al., 2000; Corbetta et al., 2000; Driver and Vuilleumier, 2001; Hilgetag et al., 2001; Vallar, 2001; Bjoertomt et al., 2002; Mort et al., 2003; Kincade et al., 2005), whereas the role of the dorsal PPC subregions, especially the superior parietal lobule (SPL) in visuospatial attention has not been well studied.

Mounting lesion-based studies have found that deficit in ventral inferior parietal lobule (IPL) and temporo-parietal junction (TPJ) caused visuospatial neglect (Friedrich et al., 1998; Bartolomeo and Chokron, 2002; Mort et al., 2003; Han et al., 2004; Verdon et al., 2010), and only few studies observed lesion in dorsal PPC subregion, SPL, causing visuospatial neglect (Gillebert et al., 2011; Vandenberghe et al., 2012). However, evidence from functional neuroimaging studies has consistently revealed that the SPL is involved in visuospatial attention (Fink et al., 2000, 2001; Szczepanski et al., 2010). In addition, a recent study using intraoperative electrical stimulation which is considered to be the gold standard to determine the brain function in awake patients during brain tumor surgery showed that stimulation of the right posterior SPL resulted in visuospatial neglect (Vallar et al., 2014). The discrepancy between the neuropsychological findings and the findings obtained from brain damaged patients raises the question what’s the role of posterior SPL in visuospatial attention.

To determine the relationship between brain and behavior, TMS technique can be used to induce a transient interruption of normal brain activity in a relatively restricted area of the brain to directly and non-invasively assess whether the brain area is involved in a specific cognitive function. Thus, TMS provides a unique opportunity to study brain-behavior relations in healthy humans (Pascual-Leone et al., 1999, 2000; Walsh and Cowey, 2000). In the current study, we used TMS to induce the temporal virtual lesions in bilateral posterior SPL to determine the role of SPL in visuospatial attention.

Visuospatial attention is considered to be primarily controlled by the dorsal and ventral fronto-parietal attention network (Corbetta and Shulman, 2002). The asymmetric dynamic balance between fronto-parietal networks in the two hemispheres is considered to result in the functional lateralization of visuospatial ability (Corbetta and Shulman, 2011; Koch et al., 2011; Thiebaut de Schotten et al., 2011). In addition to fronto-parietal network, lesion-based studies in neglect patients have proposed the unbalanced interhemispheric interactions between bilateral PPC to account for this hemispheric specialization of visuospatial function (Kinsbourne, 1977, 1993; Koch et al., 2011). The existing evidence indicated that the functional asymmetry of visuospatial attention is substrated by brain networks. Therefore, we hypothesized that the lateralization of visuospatial attention may be due to asymmetric anatomical connectivity between the bilateral posterior SPLs with their involved fronto-parietal network and the contralateral PPC.

In our current study, we aimed to directly test whether posterior SPL participates in visuospatial attention in healthy humans, and whether the asymmetry of visuospatial attention exists in SPL using TMS technique. Furthermore, we used diffusion magnetic resonance imaging (MRI) to further investigate the neuroanatomical basis of asymmetry of visuospatial attention. We firstly defined the posterior SPL on the basis of the SPL atlas constructed with different anatomical connectivity patterns in our previous study (Wang et al., 2015). Secondly, repetitive TMS was applied separately to the right and left posterior SPL to investigate the role of the posterior SPL in visuospatial attention. Finally, anatomical connectivity mapping and correlation analyses were used to determine the relationship between anatomical connections and visuospatial attention performances.

## Materials and Methods

### Subjects

Considering the possible sex-based differences in visuospatial ability (Torres et al., 2006; Ingalhalikar et al., 2014), in this study, 16 male, healthy, right-handed subjects (mean age = 18.8 years, range: 18–21 years) were recruited via advertisement. None of the participants had ever suffered from any psychiatric or neurological disease, and none had previous experience with participating in TMS measurements nor had any contraindications for MRI scanning. All subjects signed an informed consent form approved by the local Research Ethics Committee of University of Electronic Science and Technology of China. All the experiments and the methods were performed in accordance with the approved guidelines and regulations, and all experimental protocol were also approved by the Research Ethics Committee of University of Electronic Science and Technology of China.

### MRI Data Acquisition

Before the TMS experiment, all 16 subjects were scanned using a 3.0 Tesla GE MR Scanner. DTI data, which included 64 images with non-collinear diffusion gradients (b = 1000 s/mm^2^) and three non-diffusion-weighted images (b = 0 s/mm^2^), were collected. From each participant, 75 slices were collected with an acquisition matrix = 128 × 128, flip angle (FA) = 90°, voxel resolution: 2 × 2 × 2 mm^3^, and no gap. Sagittal 3D T1-weighted images were also acquired (TR/TE = 8.16/3.18 ms; inversion time = 800 ms; FA = 7°; FOV = 256 × 256 mm; matrix = 256 × 256; slice thickness = 1 mm, no gap; 188 sagittal slices).

### DTI Data Preprocessing

The MRI data were visually inspected for obvious artifacts arising from subject motion and instrument malfunction. Eddy currents and head motions were corrected using FMRIB’s Diffusion Toolbox (FSL 4.0^1^). Skull-stripped T1-weighted images of each subject were co-registered to the subject’s non-diffusion-weighted image (b = 0 s/mm^2^) using the statistical parametric mapping (SPM8) package^2^. Then the registered T1 images (rT1) obtained in diffusion space were transformed to the Montreal Neurological Institute’s colin27 structural template. Finally, an inverse transformation was performed to transform the seed and target masks into the diffusion space for each subject.

### Definition of Posterior SPL for TMS

**Figure 1:**
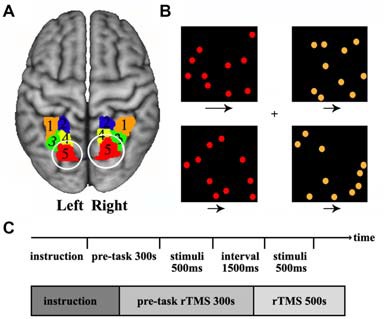
**Definition of the posterior superior parietal lobule (SPL) and the visual search task. (A)** The posterior SPL was defined using a connectivity-based parcellation approach. The maximum probability map for the SPL subregions was created and the most posterior subregions (red, label 5) in left and right SPL were selected as the target brain sites of transcranial magnetic stimulation (TMS). The labels 1–5 is the five subregions in left and right SPL. **(B)** The stimulus displayed during a target-present color task trial. The subjects searched the visual display for targets defined by a conjunction of color and motion. Four square windows were used in the experiment. Each window contained 10 dots (orange or red) that moved at two speeds (fast or slow) during the experiment, and the arrows under each window specify the two speeds at which the dots could move. **(C)** The time courses of the visual stimuli and repetitive transcranial magnetic stimulation (rTMS) stimuli. The rTMS stimuli were applied for 300 s before behavioral task start and continued until the task finished.

The previous task-based functional neuroimaging studies have showed that visuospatial attention primarily activated the posterior SPL (Corbetta et al., 1995; Rushworth et al., 2001; Vandenberghe et al., 2001; Yantis et al., 2002; Szczepanski et al., 2010). Recently, we combined connectivity-based parcellation approach and meta-based behavioral domain analyses further demonstrated that the most posterior SPL subregion was predominantly involved in visuospatial attention processing (Wang et al., 2015). Thus, we selected the bilateral most posterior SPL subregion as the TMS targets according to our previously constructed SPL atlas (Wang et al., 2015) in the current study (Figure 1A).

### TMS on the SPL Subregion

#### Visuospatial Attention Task

The spatial attention task which was adopted in our study has been used in a previous study (Corbetta et al., 1995). A series of stimuli were displayed during a target-present color task trial. The subjects searched a visual display for targets defined by a conjunction of color and motion. In the task, participants saw a display consisting of four square windows containing moving colored dots. Each window appeared at the vertex of an imaginary square, with a fixation cross at the center of the display. Each window was centered at an eccentricity of 4°, and contained 10 dots, where each dot was 30 mm in diameter. The direction of dot motion (right or left) varied randomly over trials. During the experiment, the colored dots in each window moved at two speeds (slow or fast: 3° or 10° per second), and a speed and color were randomly assigned to each window. The arrows under each window specify the two speeds at which the dots could move. The luminance of each dot was 30 cd/m^2^, and the luminance of the background was 22 cd/m^2^. The “target” condition occurred when red dots moving at a fast speed appeared in a window, and the other conditions were “non-target”. When the target dots appeared, the participants were instructed to press the right hand button. When a non-target condition appeared, the participants were instructed to press the left hand button. Each display was presented for 500 ms, followed by a 1500 ms interval for pressing the response key (Figure 1B). Totally, 250 stimuli (150 targets and 100 non-targets) were presented over a period of 500 s (Figure 1C). The experiment was a block design containing three conditions: sham stimulus, stimulus to the left SPL, and stimulus to the right SPL. The TMS were synchronized with the visual stimulus, so when moving dots were presented at the computer display, TMS was simultaneously applied on the skull.

#### TMS on the Right or Left Posterior SPL Subregion

The most posterior subregion, Cluster 5 (red/label 5), was selected as the target brain area for TMS (Figure 1A). The MNI center coordinate for the left and right posterior SPL are: L5 [−20, −70, 56] and R5 [20, −71, 50]. Before TMS, we transformed the posterior SPL subregions’ mask obtained by connectivity-based parcellation into each individual structural image (T1 space) and marked as the target sites. Frameless stereotaxy was applied to identify the exact posterior parietal subregion, and individual MRI-based TMS neuronavigation allowed for a precise localization of the target sites. Stimulation frequency was 1 Hz to reduce cortical excitability and an online repetitive transcranial magnetic stimulation (rTMS) paradigm was selected to eliminate the possibility of a change in the degree of virtual lesion effect as time passes. Before the behavioral test, the rTMS was applied for 300 s to induce a sufficient virtual lesion. After the pre-task rTMS, we asked the participants to perform a visuospatial attention test. The participants underwent two event-related TMS sessions, one on the right and the other on the left posterior SPL, separated by an interval of 1 week to exclude the previous TMS effect. To generate the magnetic pulses, we used a Magstim Rapid stimulator (The Magstim Company Limited, Whitland, UK) with a standard 70 mm figure of eight coil. The intensity of the magnetic stimulation was set separately for each individual at their motor threshold which was determined by visually detecting twitch in the contralateral hand when a stimulus was applied to the motor region. The spatial attention task was simultaneously executed by applying TMS to the posterior SPL of each participant. As a control, sham stimulation was applied with a sham coil. The reaction time and accuracy which were used to characterize the behavioral performance were recorded for further analysis. In order to evaluate the TMS effects of sham stimulation, TMS on left or TMS on right posterior SPL, the analysis of variance (ANOVA) was first performed. Next, paired two-tailed *t*-tests were performed on the reaction times for sham stimulation, TMS on left, and TMS on right. The false discovery rate (FDR) was used to identify the significant differences with *p* < 0.05.

#### Laterality Index

In this study, we employed the laterality index (LI; see Equation 1) which was utilized in a previous study to describe the asymmetry of the posterior SPL with respect to visuospatial functions and anatomical connections (Steinmetz, 1996; Tomasi and Volkow, 2012). The LI was defined as follows: Positive LI values indicate rightward asymmetry and negative LI values indicate leftward asymmetry.

(1) LI=(R−L)/(R+L)

### Anatomical Connections Mapping

#### Target Masks Definition

Many previous studies have demonstrated that the visuospatial attention is controlled by fronto-parietal network through superior longitudinal fasciculus (SLF) I, the SLF II, and the extreme capsule (EmC; Thiebaut de Schotten et al., 2005; He et al., 2007; Thiebaut de Schotten et al., 2011). The three white matter pathways can be reconstructed with the target masks of superior frontal gyrus (SFG), middle frontal gyrus (MFG), and inferior frontal gyrus (IFG), respectively (Thiebaut de Schotten et al., 2011). In our current study, to map the different fronto-parietal anatomical pathways, we defined three subregions including SFG, MFG, and IFG in the frontal cortex as the target brain areas. The target masks were delineated using the Harvard-Oxford atlas provided by FSL Software. The SFG, MFG, and IFG were each extracted using a minimum probability of 25% to get the white matter fibers which connected the posterior SPL with these brain areas. Furthermore, the PPC including the SPL and the IPL was also delineated with a minimum probability of 25%. Then, all these masks were transformed to diffusion space to obtain the white matter pathways which connected the posterior SPL to each frontal subregion.

#### Mapping the Anatomical Connections between the SPL and the Ipsilateral Frontal Subregions

To detect whether unbalanced interhemispheric interaction between the bilateral fronto-parietal networks results in asymmetric visuospatial ability, we studied whether asymmetry of visuospatial attention was related to asymmetric anatomical connections between the bilateral posterior SPLs with their ipsilateral frontal subregions. To explore the influences of different fronto-parietal pathways on the asymmetric visuospatial functions, we subdivided the frontal cortex into three subareas including the SFG, MFG, and IFG in MNI space and transformed all the masks into diffusion space to reconstruct the white matter pathways. Then, we used probabilistic fiber tracking to detect the fiber matter pathway between the posterior SPL and each frontal subregion in the same hemisphere. When tracking a specific white matter pathway between the posterior SPL and the frontal subregion, the other two subregions were defined as exclusion masks to exclude the connections passing from those two areas to the target area. The anatomical connectivity strength between posterior SPL and each frontal subarea was characterized using connectivity probability between each pair of regions in our current study. In order to calculate the connectivity probability, probabilistic fiber tracking was performed using FSL Software. The probability distributions were computed for two fiber directions at each voxel (Behrens et al., 2007). To estimate the connectivity probability, probabilistic tractography was applied by sampling 5000 streamline fibers per voxel. The connectivity probability from the seed voxel *i* to another voxel *j* was defined by the number of fibers passing through voxel *j* divided by the total number of fibers sampled from voxel *i*. The idea of connectivity between voxels could be extended from the voxel level to the regional level. For a seed region, 5000× *n* streamlines were sampled (5000 streamlines for each voxel), where *n* is the number of voxels in the seed region. The number of fibers passing through a given region divided by 5000× *n* is computed and defined as the connectivity probability from the seed region to the target region (Gong et al., 2009). Subsequently, the paired two-tailed *t*-tests were performed and the FDR correction (*p* < 0.05) was used to determine the significant differences in anatomical connections between hemispheres. Finally, the LI of the anatomical connections between the right and left posterior SPL with each frontal subregion were calculated. Correlation analyses between the LI of an anatomical connection and the LI of the reaction time were applied to determine their relationship. FDR correction and *p* < 0.05 was set for significance.

#### Anatomical Connections Mapping between the SPL and the Contralateral PPC

In order to investigate whether the asymmetry of visuospatial attention is related to asymmetrical anatomical connections between the posterior SPL and the contralateral PPC, the anatomical connectivity probability between the posterior SPL and the contralateral PPC in each subject were also calculated. Then, a paired two-tailed *t*-test on the connectivity probability of the left and right posterior SPL was performed, and FDR correction (*p* < 0.05) was used to identify the significance. The LI of anatomical connectivity probability of the left and right posterior SPL was calculated, and correlation analyses between the LI of the connectivity probability and the LI of the reaction time were also executed. The significance threshold was set at *p* < 0.05 (FDR correction).

## Results

### Behavioral Tests

**Figure 2:**
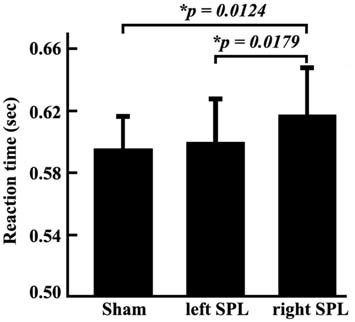
**Reaction times of behavioral tests in the three conditions.** The experiment was a block design containing three conditions: sham stimulus; stimulus to the left SPL; and stimulus to the right SPL. During each condition, the reaction time of each subject was recorded. Then, mean reaction times and standard error of the mean in each condition was calculated. The paired two-tailed *t*-tests were performed on the reaction time, and the threshold was set at *p* < 0.05 for significance.

In the TMS experiments, the reaction times and accuracy for each subject were respectively recorded. We did not find the significant differences between accuracy when TMS were separately applied in the left or right posterior SPL (accuracy: left, 93.16%, right, 92.38%). The ANOVA identified significant differences of reaction times under the conditions of sham stimulation, TMS on left SPL, and TMS on right SPL (F-value: 8.59, *p*-value: 0.6924 × 10^−3^). Compared with sham stimulation, TMS applied on right posterior SPL induced increment of reaction time (*p* = 0.0124). In addition, the reaction times also differed when TMS separately applied over the left and right posterior SPLs (*p* = 0.0179). The reaction time was significantly longer when the stimuli were applied in the right SPL than when they were applied in the left SPL (Figure 2). The paired *t*-test also revealed that significantly longer reaction times were observed in the spatial attention task test when the stimuli were applied to the right SPL (0.0153 ± 0.0057 s; *p* = 0.0179; Figure 2).

### Anatomical Connections Mapping between the SPL and the Ipsilateral Frontal Subregions

**Figure 3:**
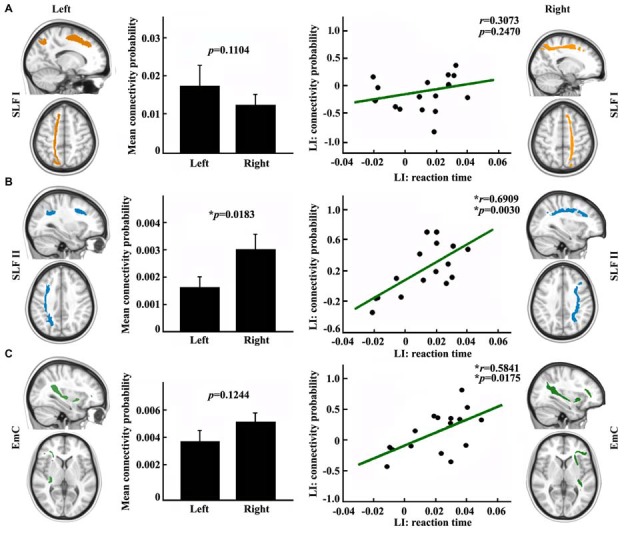
**Anatomical connections mapping analyses between the posterior SPL and the frontal subregions. (A)** The white matter pathway of superior longitudinal fasciculus (SLF) I between the posterior SPL and the superior frontal gyrus (SFG) is shown in the upper panel. The mean anatomical connectivity probability and the standard error and correlation analysis for the laterality index (LI) of anatomical connections of the posterior SPL to the SFG and the LI of the reaction time are also portrayed in the upper panel. **(B)** The white matter pathway of SLF II between the posterior SPL to the middle frontal gyrus (MFG), the mean anatomical connectivity probability and the correlation analysis for the LI of the anatomical connections of the posterior SPL to the MFG and the LI of the reaction time are delineated in the middle panel. **(C)** The fibers of extreme capsule (EmC) between the posterior SPL to the inferior frontal gyrus (IFG), mean anatomical connectivity probability, and the correlation analysis for the LI of the anatomical connections of the posterior SPL to the IFG and LI of the reaction time are delineated in the lowest panel. The unit of reaction time is second (s). * Represents the statistically significant difference; false discovery rate (FDR) corrected, *p* < 0.05 for significance.

In order to explore the influences of different fronto-parietal pathways on the asymmetry of visuospatial attention, we used different frontal subregions to reconstruct the different fronto-parietal white matter pathways. The different white matter pathways between the posterior SPL and frontal subregions were identified. The main fiber pathways that connected the posterior SPL with the SFG and the posterior SPL with the MFG are the SLF I (Figure 3A) and the SLF II (Figure 3B), respectively, whereas the main fiber pathway that connected the posterior SPL with the IFG is the EmC (Figure 3C). Subsequently, paired *t*-tests and correlation analyses were respectively employed to investigate the differences between the hemispheres in the anatomical connections of the posterior SPL to each frontal subregion and to study whether the asymmetric anatomical connections were related to the asymmetry of visuospatial attention. The paired *t*-tests analyses revealed that significant differences between the hemispheres in the anatomical connections were found between the posterior SPL and the ipsilateral MFG (Figure 3B). Correlation analyses unraveled that the asymmetric connections of the IFG and MFG with the posterior SPL were significantly related to the asymmetry of the visuospatial attention (Figures 3B,C).

### Anatomical Connectivity Mapping between the SPL and the Contralateral PPC

**Figure 4:**
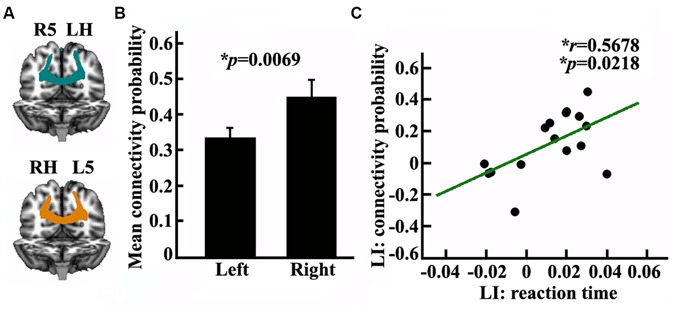
**Anatomical connectivity analyses between the posterior SPL and the contralateral posterior parietal cortex (PPC). (A)** The white matter pathway of corpus callosum (CC) between the posterior SPL and the contralateral PPC is shown in the left panel, LH: left hemisphere, RH: right hemisphere, L5: the left posterior SPL subregion labeled #5, R5: the right posterior SPL subregion labeled #5. **(B)** The middle panel shows the mean anatomical connectivity probability and the standard error for the left and right posterior SPL to the contralateral PPC. **(C)** The correlation analyses for the LI of anatomical connectivity probability and the LI of the reaction time. The unit of reaction time is second (s). *Represents the statistically significant differences; FDR corrected, *p* < 0.05.

In this study, we measured the anatomical connectivity strength between the posterior SPL and the contralateral PPC using probabilistic tracking. The main fiber pathway which connected the posterior SPL and contralateral PPC was the posterior corpus callosum (CC; Figure 4A). The connectivity probability of the bilateral posterior SPL to the contralateral PPC was calculated for each subject in diffusion space. There was a significantly different connectivity probability for the two hemispheres between the SPL and the contralateral PPC (*p* = 0.0069; Figure 4B). The correlation analysis indicated that the asymmetric anatomical connections of the SPL to the contralateral PPC were significantly related to the asymmetry of visuospatial attention (Figure 4C).

## Discussion

Our goal was to assess how the posterior SPL controls the right hemisphere dominance in visuospatial attention. Furthermore, we used diffusion MRI and rTMS to investigate the neuroanatomical basis for the functional specification. Anatomical connectivity mapping analyses revealed that the asymmetry of visuospatial attention was mediated by both the fronto-parietal network and the contralateral PPC network via the SLF II, EmC and posterior CC. Our study shows potential for providing the anatomical basis for how the SPL controls rightwardly lateral visuospatial attention.

The fronto-parietal networks were mainly linked by white matter pathways of SLF. Makris et al. (2005) identified the four subcomponents of SLF in the human brain using *in vivo* diffusion tensor imaging. Thiebaut de Schotten et al. (2011) used diffusion MRI to delineate the SLF I and SLF II *in vivo* and revealed only SLF II plays an key role in visuospatial attention. In addition, attention was controlled by dorsal and ventral attention networks in which the core brain areas were superior, middle, and IFG (Corbetta and Shulman, 2002). Therefore, in our current study, we used the superior, middle, and IFG as seed areas to map the fronto-parietal anatomical connections and revealed that both the SLF II and EmC were closely related to lateralization of visuospatial attention.

Lateralization of visuospatial attention was firstly revealed by clinical study in patients after stroke (Stone et al., 1993). However, different studies have observed different effects on visuospatial attention after damage to SPL (Friedrich et al., 1998; Gillebert et al., 2011; Vandenberghe et al., 2012). The discrepancy may be mainly caused by inconsistent damaged area, volume and properties across patients. Although neuroimaging-based studies have uncovered that the SPL participated in visuospatial attention (Corbetta and Shulman, 2002; Fan et al., 2005), neuroimaging approach cannot determine whether a specific brain area is crucial for a specific cognitive function. Contrarily, TMS technique can model virtual lesion in a specific brain area. Above all, TMS can guarantee the consistency for location, volume and property of lesion across subjects (Pascual-Leone et al., 2000; Walsh and Cowey, 2000; Calvo-Merino and Haggard, 2004; Abler et al., 2005; Sandrini et al., 2011). Thus, TMS-based study can better reveal the neural mechanism for human cognitive function. Here, using TMS approach, our findings demonstrate that SPL participated in visuospatial attention processing, which may resolve the debate on this open problem.

Functional neuroimaging studies suggested that visuospatial attention is mainly controlled by dorsal and ventral fronto-parietal networks. The dorsal fronto-parietal network is bilaterally organized, whereas the ventral fronto-parietal network is largely lateralized to the right hemisphere (Corbetta and Shulman, 2002). The dorsal fronto-parietal network is mainly involved in cognitive selection of sensory information and guiding eye movements (Szczepanski et al., 2013). In our current study, we did not find the asymmetric anatomical connections between SPL and SFG. This finding indicated that the dorsal fronto-parietal network is equally contributed to cognitive selection of sensory information and response. Contrarily, the white matter pathway of the EmC, which connected the posterior SPL and the ipsilateral IFG, was found to be closely correlated to visuospatial attention performance in our study suggesting that the fiber pathway of the EmC might mediate the right hemispheric dominance in visuospatial attention. Previous task-based fMRI studies reported that the posterior SPL primarily participated in attention shifting/switching and the IFG primarily participated in cognitive control, task switching and reorienting attention (Corbetta et al., 1993, 1995, 2008; Rushworth et al., 2001; Neubert et al., 2014), which indicated that the EmC plays an important role in controlling attention, especially in task switching and reorienting attention. Therefore, the EmC pathway constitutes an anatomical substrate for the orientating network (Fan et al., 2005). Furthermore, the EmC has been found to connect the SPL with the IFG by passing through the temporoparietal junction area (TPJ; Makris and Pandya, 2009). Both SPL and TPJ were found to be involved in visuospatial attention shifting task, and the SPL was primarily activated during attention shifting, whereas the TPJ was mainly activated during maintaining of attention (Rushworth et al., 2001; Vandenberghe et al., 2001). Thus, our tractographic results with the previous task-based functional neuroimaging findings might account for why a TPJ deficit will cause neglect (Mort et al., 2003; Corbetta et al., 2005).

The interaction between dorsal and ventral attention networks were considered to be mediated by MFG which is functionally connected with both dorsal and ventral attention networks (He et al., 2007). In our study, we found that the right hemispheric dominance in visuospatial attention performance was closely related to interhemispheric asymmetric anatomical connections between posterior SPL and the MFG connected by the fiber pathway of the SLF II (Makris et al., 2005). This finding was supported by previous diffusion MRI-based studies. Thiebaut de Schotten et al. (2011) found that asymmetric volume of the SLF II was related to asymmetric visuospatial attention and that larger volumes of axons in the parieto-frontal network transfer information at faster conduction speeds. Another TMS-based study also revealed that the fractional anisotropy (FA) values of the SLF II were also correlated with visuospatial attention performance (Koch et al., 2011). These studies collectively demonstrated MFG plays an important role in visuospatial attention. Thus, MFG may be mainly involved in coupling the dorsal and ventral network to coordinate the interaction between the two networks. Furthermore, the anatomical connectivity mapping results in our study seem to indicate that the MFG coordinates the ventral attention network for reorienting attention through the posterior SPL via the SLF II and EmC.

Previous studies proposed that interhemispheric competition or imbalanced interaction between the hemispheric PPCs underlie the brain asymmetry in visuospatial abilities (Kinsbourne, 1977, 1993). Here, we used TMS technique and revealed functional asymmetry of the bilateral posterior SPL in visuospatial attention, a finding which was consistent with a previous functional neuroimaging finding (Corbetta et al., 1993). The subsequent anatomical connectivity mapping found asymmetric white matter pathways of the bilateral posterior SPLs with the contralateral PPC. The identified asymmetric white matter pathway of the posterior CC to the contralateral PPC was consistent with the previous studies (Hofer and Frahm, 2006; Koch et al., 2011). However, the exact role of the posterior CC in human cognitive ability has not been well documented. The connectivity mapping and correlation analysis in our study indicated that the asymmetric posterior CC connecting the bilateral PPC played an important role in visuospatial attention. The stronger connections of the right SPL to the contralateral PPC than that of the left SPL supported the dominant role of the right SPL in visuospatial attention. In addition, our findings suggest that asymmetry of visuospatial attention was controlled by distributed networks including the fronto-parietal network and the bilateral parietal network. We also confirmed that the interhemispheric parietal network was significantly related to the control of the right hemisphere dominance in visuospatial attention via the posterior CC.

## Author Contributions

YW, JW, YW, KZ, and TJ designed the study. YW, YZ, DZ, JZ, MR, and HW performed the experiments. YW and JW analyzed the data. YW, JW and TJ wrote the article.

## Conflict of Interest Statement

The authors declare that the research was conducted in the absence of any commercial or financial relationships that could be construed as a potential conflict of interest. The reviewer MCP and the handling Editor declared their shared affiliation, and the handling Editor states that the process nevertheless met the standards of a fair and objective review.

## Footnotes

^1^http://www.fmrib.ox.ac.uk/fsl

^2^http://www.fil.ion.ucl.ac.uk/spm
